# Efficacy of Tamoxifen Metabolites in Combination with Colistin and Tigecycline in Experimental Murine Models of *Escherichia coli* and *Acinetobacter baumannii*

**DOI:** 10.3390/antibiotics13050386

**Published:** 2024-04-24

**Authors:** Soraya Herrera-Espejo, Andrea Vila-Domínguez, Tania Cebrero-Cangueiro, Younes Smani, Jerónimo Pachón, Manuel E. Jiménez-Mejías, María E. Pachón-Ibáñez

**Affiliations:** 1 Unidad Clínica de Enfermedades Infecciosas, Microbiología y Parasitología, Instituto de Biomedicina de Sevilla (IBiS), Hospital Universitario Virgen del Rocío/CSIC/Universidad de Sevilla, 41013 Sevilla, Spain; 11.sorayaherrera.2b@gmail.com (S.H.-E.); andrea.vila.dguez@gmail.com (A.V.-D.); tany5_91@hotmail.com (T.C.-C.); mej-mejias@telefonica.net (M.E.J.-M.); mpachon-ibis@us.es (M.E.P.-I.); 2 Centro Andaluz de Biología del Desarrollo, Universidad Pablo de Olavide/Consejo Superior de Investigaciones Científicas/Junta de Andalucía, 41013 Sevilla, Spain; y_smani@hotmail.com; 3 Departamento de Biología Molecular e Ingeniería Bioquímica, Universidad Pablo de Olavide, 41013 Sevilla, Spain; 4 Instituto de Biomedicina de Sevilla (IBiS), Hospital Universitario Virgen del Rocío/CSIC/Universidad de Sevilla, 41013 Sevilla, Spain; 5 Departamento de Medicina, Facultad de Medicina, Universidad de Sevilla, 41009 Sevilla, Spain; 6 CIBER de Enfermedades Infecciosas (CIBERINFEC), Instituto de Salud Carlos III, 28220 Madrid, Spain

**Keywords:** repurposing, N-desmethyltamoxifen metabolite, colistimethate sodium, tigecycline, experimental murine models, *Acinetobacter baumannii*, *Escherichia coli*

## Abstract

This study aimed to evaluate the potential of tamoxifen and N-desmethyltamoxifen metabolites as therapeutic agents against multidrug-resistant *Escherichia coli* and *Acinetobacter baumannii,* using a repurposing approach to shorten the time required to obtain a new effective treatment against multidrug-resistant bacterial infections. Characterisation and virulence studies were conducted on *E. coli* (colistin-susceptible C1-7-LE and colistin-resistant MCR-1+) and *A. baumannii* (tigecycline-susceptible Ab#9 and tigecycline-resistant Ab#186) strains. The efficacy of the metabolite mix (33.3% each) and N-desmethyltamoxifen in combination with colistimethate sodium (CMS) or tigecycline was evaluated in experimental models in mice. In the pneumonia model, N-desmethyltamoxifen exhibited significant efficacy against Ab#9 and both *E. coli* strains, especially *E. coli* MCR-1+ (−2.86 log_10_ CFU/g lungs, −5.88 log_10_ CFU/mL blood, and −50% mortality), and against the Ab#186 strain when combined with CMS (−2.27 log_10_ CFU/g lungs, −2.73 log_10_ CFU/mL blood, and −40% mortality) or tigecycline (−3.27 log_10_ CFU/g lungs, −4.95 log_10_ CFU/mL blood, and −50% mortality). Moreover, the metabolite mix in combination with both antibiotics decreased the bacterial concentrations in the lungs and blood for both *A. baumannii* strains. In the sepsis model, the significant efficacy of the metabolite mix was restricted to the colistin-susceptible *E. coli* C1-7-LE strain (−3.32 log_10_ CFU/g lung, −6.06 log_10_ CFU/mL blood, and −79% mortality). N-desmethyltamoxifen could be a new therapeutic option in combination with CMS or tigecycline for combating multidrug-resistant GNB, specifically *A. baumannii*.

## 1. Introduction

Infections caused by Gram-negative bacilli (GNB) remain a significant global health burden [1], contributing to a high number of hospitalisations, with an estimation in 2019 of 1.27 global million deaths due to drug-resistant bacterial pathogens [2], together with the increased consumption of healthcare resources [3]. It is estimated that approximately 50% of intensive care unit (ICU) patients are infected with GNB [4]. The emergence of multidrug-resistant (MDR) GNB, such as *Escherichia coli* and *Acinetobacter baumannii*, further complicates the management of these infections, emphasizing the need for novel and effective treatment strategies [5].

Both microorganisms are prevalent pathogens responsible for nosocomial infections, closely associated with the development of antimicrobial resistance (AMR) [6,7]. The misuse and overuse of antimicrobials have accelerated this process [1]. Consequently, these bacteria have developed resistance to a wide range of antibiotics, including polymyxins and tetracyclines [8]. The recent joint report by the European Centre for Disease Prevention and Control (ECDC) and the World Health Organization (WHO)/Europe in April 2023 highlighted alarmingly high percentages of resistance to last-line antibiotics, such as carbapenems [9]. Additionally, colistin, a last-resort treatment for life-threatening infections, has become ineffective due to detected resistance in various countries and regions [10].

In a recent study, *A. baumannii* isolates showed a decreased trend in relation to susceptibility to colistin, from 99.7% to 86.7% and from 97.8% to 91.1% in Asia and Europe, respectively. Notably, isolates from Europe showed the highest colistin minimum inhibitory concentration, where 90% of the isolates were inhibited (MIC_90,_ 2 mg/L). Throughout the years 2020 and 2021, isolates from Europe had the lowest colistin-susceptible rates, namely 92.0% and 92.6%, respectively [11].

In spite of the efforts focused on developing new antimicrobials, progress has been limited, with only two new drugs for bacterial infection treatment approved by the Food and Drug Administration (FDA) between 2020 and 2023, targeting *Helicobacter pylori* [12] and the prevention of catheter-related bloodstream infections in adults with kidney failure [13]. Alternative approaches have been widely studied, including the conjugation of antimicrobial peptides [14], programs to optimise existing antibiotic use [15], clustered regularly interspaced short palindromic repeats (CRISPR) technology [16], and the use of vaccines and other preventative measures against infections [17].

Drug repositioning has demonstrated promising therapeutic potential for combating bacterial infections [18]. Anticancer drugs, such as mitomycin C and mitotane, have shown antimicrobial activity against *A. baumannii* and *E. coli* [19]. Tamoxifen, a selective oestrogen receptor modulator, exhibits bactericidal properties through the regulation of immune cell traffic after GNB infection and an effective reduction in the hyper-inflammation caused by sepsis and bacterial burdens in animal tissue and fluids [20]. Tamoxifen is metabolised in the liver by cytochrome P450 into two primary metabolites, 4-hydroxytamoxifen and N-desmethyltamoxifen, and a secondary metabolite, endoxifen [21]. Approximately 92% of tamoxifen is catalysed to N-desmethyltamoxifen [22], which is the major circulating metabolite in plasma [23]. Previous data obtained in our laboratory have shown that these metabolites also exhibit bactericidal activity against MDR GNB, including *E. coli* and *A. baumannii* [24], but at non-humanised dosages [20,24]. 

Based on these findings, we aimed to evaluate the potential efficacy of tamoxifen metabolites at humanised dosages, specifically N-desmethyltamoxifen against *E. coli* and *A. baumannii* in commonly used experimental murine models of peritoneal sepsis and pneumonia [25,26], both as monotherapy and in combination with antimicrobials currently used in clinical practice, and to search for valuable data useful for difficult-to-treat infections caused by multidrug-resistant strains.

## 2. Results

### 2.1. Antimicrobial Activity

The MIC/minimum bactericidal concentration (MBC) of tamoxifen and its three metabolites, the metabolite mix, colistimethate sodium (CMS), and tigecycline are detailed in Table 1. Regarding experimental drugs, the metabolite mix had the lowest MIC, 4 mg/L, for the four strains, and N-desmethyltamoxifen had an MIC of 128 mg/L for both *E. coli* strains and 16 and 64 mg/L for *A. baumannii* Ab#9 and Ab#186, respectively.

### 2.2. Time–Kill Curves

**Figure 1:**
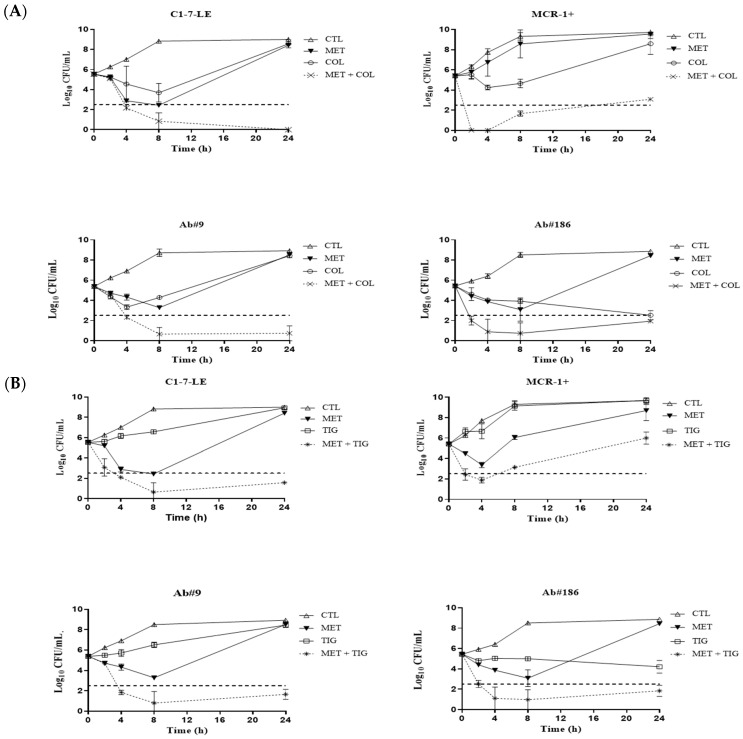
Time–kill curves of colistin (**A**) and tigecycline (**B**) alone and combined with tamoxifen and metabolite mix, at MIC concentrations, against the *Escherichia coli* C1-7-LE (colistin-susceptible), *E. coli* MCR-1+ (colistin-resistant), *Acinetobacter baumannii* Ab#9 (tigecycline-susceptible), and *A. baumannii* Ab#186 (tigecycline-resistant) strains. Empty triangle: growth control (CTL); inverted filled triangle: tamoxifen metabolite mix (MET); empty circle: colistin (COL); cross: COL + MET; empty square: tigecycline (TIG); asterisk: TIG + MET; dotted line: bactericidal activity.

The activity of colistin and/or tigecycline alone and combined with the metabolite mix at the MICs is shown in Figure 1 and Supplementary Table S3. For the colistin-susceptible *E. coli* C1-7-LE strain, both combinations were bactericidal from 4 to 24 h, with synergistic activity in the colistin and tigecycline combinations at 24 h. For the colistin-resistant *E. coli* MCR-1+ strain, colistin in combination with the metabolite mix was synergistic from 2 h to 24 h, and the combination of tigecycline with the metabolites was synergistic from 8 to 24 h. When measured against the tigecycline-susceptible *A. baumannii* Ab#9 strain, both combinations with colistin and tigecycline were bactericidal from 4 to 24 h and synergistic from 8 to 24 h and from 4 to 24 h, respectively. Finally, for the tigecycline-resistant *A. baumannii* Ab#186 strain, colistin plus the metabolite mix was bactericidal at all of the analysed time-points and was synergistic from 2 to 24 h; moreover, tigecycline plus the metabolite mix was bactericidal and synergistic from 4 to 24 h.

**Figure 2:**
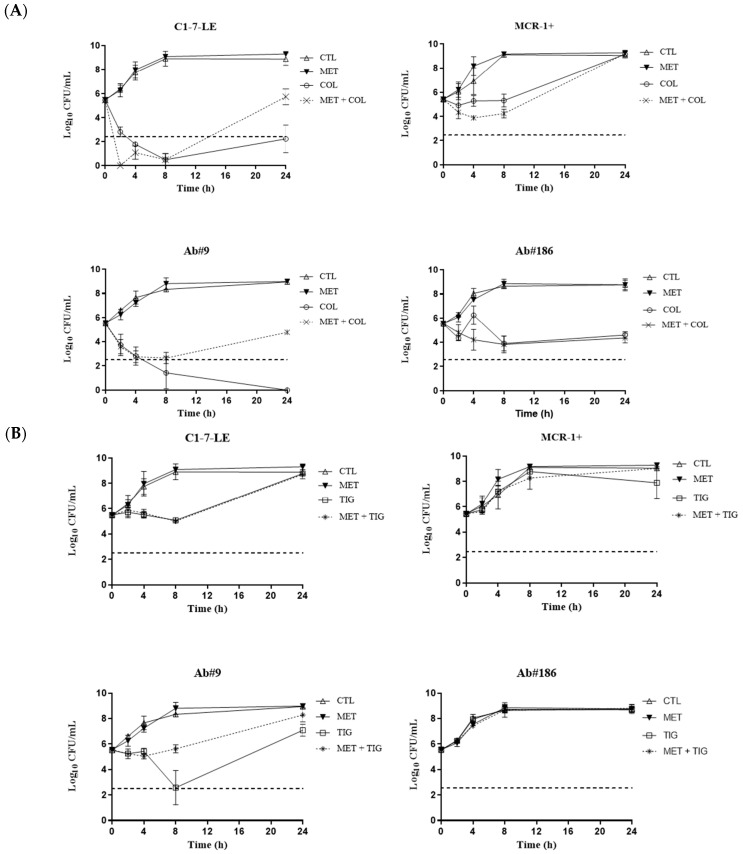
Time–kill curves of colistin (**A**) and/or tigecycline (**B**) alone and combined with tamoxifen metabolites, at plasmatic mouse C*_max_*, against *Escherichia coli* C1-7-LE (colistin-susceptible), *E. coli* MCR-1+ (colistin-resistant), *Acinetobacter baumannii* Ab#9 (tigecycline-susceptible), and *A. baumannii* Ab#186 (tigecycline-resistant) strains. Empty triangle: growth control (CTL); inverted filled triangle: tamoxifen metabolite mix (MET); empty circle: colistin (COL); cross: COL + MET; empty square: tigecycline (TIG); asterisk: TIG + MET; dotted line: bactericidal activity.

The activity of colistin and/or tigecycline alone and combined with the metabolite mix at the serum mice maximum concentration (C*_max_*) values (Table 2) was lower than that at the MICs (Figure 2 and Supplementary Table S4). For the colistin-susceptible *E. coli* C1-7-LE strain, colistin in combination with the metabolite mix was bactericidal from 2 to 8 h and synergistic at 2 h, and for the tigecycline-resistant *A. baumannii* Ab#186 strain, colistin plus the metabolite mix was synergistic at 4 h. Nevertheless, for the colistin-resistant *E. coli* MCR-1+ and tigecycline-susceptible Ab#9 strains, neither combination was bactericidal or synergistic.

Finally, we evaluated the activity of the antimicrobials combined with the major circulating metabolite, N-desmethyltamoxifen, at C*_max_* (Supplementary Table S5). Colistin in combination with N-desmethyltamoxifen was bactericidal from 4 to 24 h and synergistic at 24 h against the colistin-susceptible *E. coli* C1-7-LE strain and synergistic at 4 h for the tigecycline-resistant *A. baumannii* Ab#186 strain. There was neither bactericidal nor synergistic activity of the N-desmethyltamoxifen combinations against the colistin-resistant *E. coli* mobilised colistin resistance (MCR)-1+ strain and the tigecycline-susceptible Ab#9 strain.

### 2.3. In Vivo Studies

#### 2.3.1. Pharmacokinetics (PK) and Pharmacodynamics (PD)

The PK and PD profiles of tamoxifen and its metabolites for each strain and the PD of CMS and tigecycline are detailed in Table 2. The PK data for CMS and tigecycline were previously determined in mice [28,29]. The activity of both antimicrobials is concentration-dependent, so the area under the drug concentration–time curve over a 24 h period to the MIC (AUC_0–24_/MIC) was higher for the susceptible strains (Table 2). 

Tamoxifen at a dose of 40 mg/kg achieved a C*_max_* similar to that achieved in humans. Due to the low plasmatic concentrations of tamoxifen and its metabolites, the C*_max_*/MIC was practically negligible, and the % of ∆T/MIC was 0, in addition to the AUC_0–24_/MIC of tamoxifen for all strains. Conversely, the AUC_0–24_/MIC of N-desmethyltamoxifen showed a difference in drug exposure among strains, being up to nine times higher for the tigecycline-susceptible *A. baumannii* Ab#9 strain than for the tigecycline-resistant *A. baumannii* Ab#186 strain, and double in the case of the *A. baumannii* Ab#186 strain compared to both *E. coli* strains.

#### 2.3.2. Efficacy of the Metabolite Mix Combinations in the Peritoneal Sepsis Model

**Figure 3:**
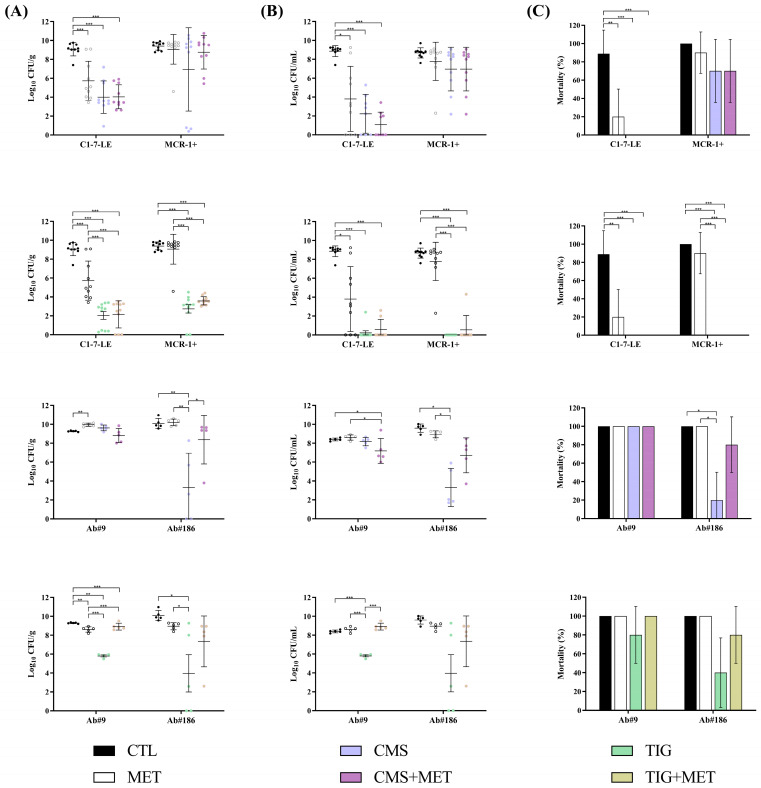
Efficacy of colistimethate sodium or tigecycline monotherapies alone and in combination with the tamoxifen metabolite mix in the peritoneal sepsis model of *Escherichia coli* C1-7-LE (n = 10, colistin-susceptible), *E. coli* MCR-1+ (n = 10, colistin-resistant), *Acinetobacter baumannii* Ab#9 (n = 5, tigecycline-susceptible), and *A. baumannii* Ab#186 (n = 5, tigecycline-resistant). Black dots and columns: control (CTL) groups; white dots and columns: metabolite mix (MET) groups; blue dots and columns: colistimethate sodium (CMS) groups; purple dots and columns: CMS in combination with MET groups; green dots and columns: tigecycline (TIG) groups; yellow dots and columns: TIG in combination with MET groups. (**A**) Efficacy of CMS and TIG monotherapies alone and in combination with MET in terms of spleen bacterial concentrations (means ± SD); (**B**) efficacy of CMS and TIG monotherapies alone and in combination with MET on blood bacterial concentrations (means ± SD); (**C**) efficacy of CMS and TIG monotherapies alone and in combination with MET in terms of mortality (means ± 95% CI). * *p* < 0.05; ** *p* < 0.01; *** *p* < 0.001.

For the colistin-susceptible C1-7-LE strain, the metabolite mix alone decreased the bacterial concentrations in lungs and blood (−3.32 log_10_ CFU/g and −6.06 log_10_ CFU/mL, *p* < 0.05) and the mortality rate (−79%, *p* < 0.05) compared to those in the control group, without having any effect against the colistin-resistant *E. coli* MCR-1+ strain. In addition, the combinations with CMS or tigecycline were not better than the CMS and tigecycline monotherapies. For both *A. baumannii* strains, i.e., tigecycline-susceptible (Ab#9) and tigecycline-resistant (Ab#186), the metabolite mix alone or in combination with CMS and tigecycline did not improve the results with respect to the controls or the antimicrobials alone in the first set of results. This prevented us from increasing the mouse sample size (Figure 3 and Supplementary Table S6) to fulfil the 3R rules, due to the insignificant efficacy obtained in this model.

#### 2.3.3. Efficacy of Metabolite Mix and N-Desmethyltamoxifen Combinations in the Experimental Pneumonia Model

**Figure 4:**
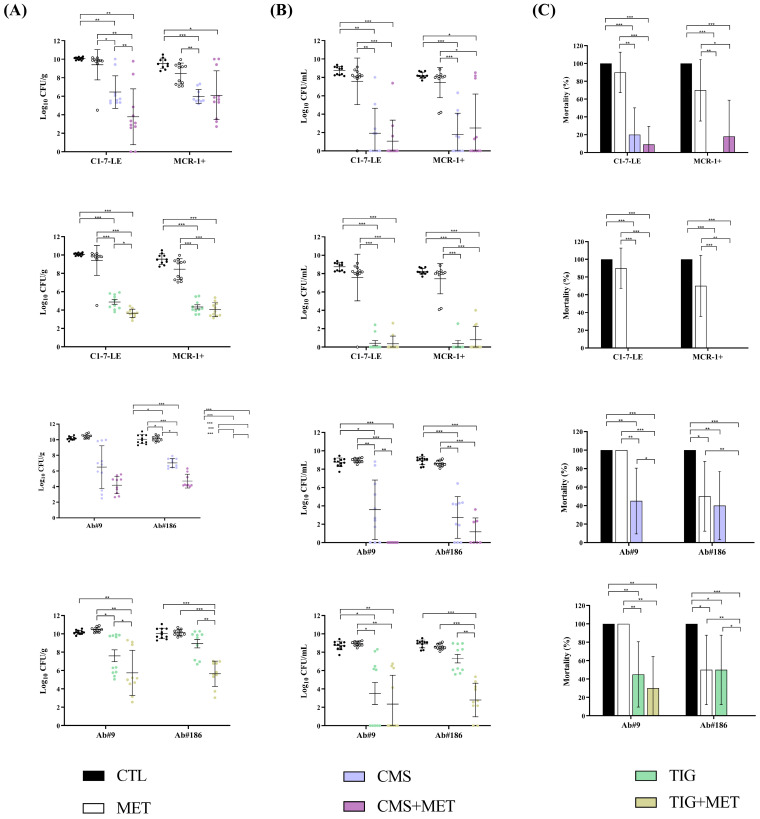
Efficacy of colistimethate sodium or tigecycline monotherapies and in combination with tamoxifen metabolite mix in the pneumonia model with *Escherichia coli* C1-7-LE (n = 10, colistin-susceptible), *E. coli* MCR-1+ (n = 10, colistin-resistant), *Acinetobacter baumannii* Ab#9 (n = 10, tigecycline-susceptible), and *A. baumannii* Ab#186 (n = 10, tigecycline-resistant). Black dots and columns: control (CTL) groups; white dots and columns: metabolite mix (MET) groups; blue dots and columns: colistimethate sodium (CMS) groups; purple dots and columns: CMS in combination with MET groups; green dots and columns: tigecycline (TIG) groups; yellow dots and columns: TIG in combination with MET groups. (**A**) Efficacy of CMS and TIG monotherapies alone and in combination with metabolite mix (MET) on lung bacterial concentrations (means ± SD); (**B**) efficacy of CMS and TIG monotherapies alone and in combination with MET on blood bacterial concentrations (means ± SD); (**C**) efficacy of CMS and TIG monotherapies alone and in combination with MET on mortality (means ± 95% CI). * *p* < 0.05; ** *p* < 0.01; *** *p* < 0.001.

In mice infected with the colistin-susceptible *E. coli* C1-7-LE and colistin-resistant *E. coli* MCR-1+ strains, the metabolite mix alone did not reduce the bacterial concentration in lungs and blood or the BSI and mortality rates compared with the control group; moreover, when combined with CMS, the combination did not reduce the bacterial lung or blood concentrations compared to CMS monotherapy (Figure 4). However, the combination of the metabolite mix with tigecycline for the colistin-susceptible C1-7-LE strain reduced the bacterial lung concentrations compared to tigecycline monotherapy (−1.24 log_10_ CFU/g, *p* < 0.05). In the *A. baumannii* pneumonia model, only the metabolite mix alone reduced the mortality rate (−50%, *p* < 0.05) compared to the control group of the tigecycline-resistant Ab#186 strain. The combination of the metabolite mix plus CMS reduced the bacterial lung and blood concentrations in the tigecycline-susceptible Ab#9 strain (−2.31 log_10_ CFU/g and −3.57 log_10_ CFU/mL, *p* < 0.05) and tigecycline-resistant Ab#186 strain (−2.30 log_10_ CFU/g and −1.56 log_10_ CFU/mL, *p* < 0.05), as well as the bacteraemia and mortality rates for the Ab#9 strain (−73% and −45%, *p* < 0.05), compared with CMS monotherapy. Similarly, the metabolite mix plus tigecycline combination reduced the bacterial lung concentrations of both the Ab#9 and Ab#186 strains (−1.85 and −3.28 log_10_ CFU/g, *p* < 0.05), as well as the blood concentration of the Ab#186 strain (−1.56 log_10_ CFU/mL, *p* < 0.05), compared with tigecycline monotherapy (Figure 4 and Supplementary Table S7). 

**Figure 5:**
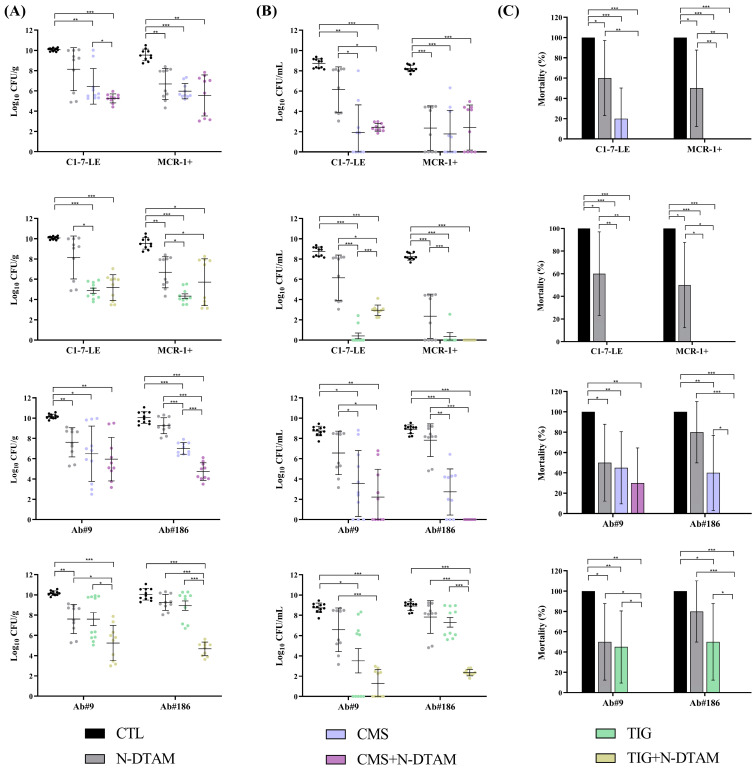
Efficacy of colistin or tigecycline monotherapies and in combination with N-desmethyltamoxifen in the pneumonia model with *Escherichia coli* C1-7-LE (n = 10, colistin-susceptible), *E. coli* MCR-1+ (n = 10, colistin-resistant), *Acinetobacter baumannii* Ab#9 (n = 10, tigecycline-susceptible), and *A. baumannii* Ab#186 (n = 10, tigecycline-resistant). Black dots and columns: control (CTL) groups; grey dots and columns: N-desmethyltamoxifen (N-DTAM) groups; blue dots and columns: colistimethate sodium (CMS) groups; purple dots and columns: CMS in combination with N-DTAM groups; green dots and columns: tigecycline (TIG) groups; yellow dots and columns: TIG in combination with N-DTAM groups. (**A**) Efficacy of CMS and TIG monotherapies alone and in combination with N-DTAM on lung bacterial concentrations (means ± SD); (**B**) efficacy of CMS and TIG monotherapies alone and in combination with N-DTAM on blood bacterial concentrations (means ± SD); (**C**) efficacy of CMS and TIG monotherapies and in combination with N-DTAM on mortality (means ± 95% CI). * *p* < 0.05; ** *p* < 0.01; *** *p* < 0.001.

The administration of N-desmethyltamoxifen alone in the pneumonia model, compared to the control group (Figure 5), reduced the mortality rate (−40%, *p* < 0.05) of the colistin-susceptible *E. coli* C1-7-LE strain, as well as the lung and blood bacterial concentrations (−2.86 log_10_ CFU/g and −5.88 log_10_ CFU/mL, *p* < 0.05) and the BSI and mortality rates (−40% and −50%, *p* < 0.05) of the colistin-resistant *E. coli* MCR-1+ strain. The combination of N-desmethyltamoxifen with CMS was better than CMS monotherapy only in relation to the reduction in the bacterial lung concentrations for the colistin-susceptible *E. coli* C1-7-LE strain (−1.19 log_10_ CFU/g, *p* < 0.05) and was better than tigecycline monotherapy in decreasing the mortality rate of the colistin-resistant *E. coli* MCR-1+ strain (−40%, *p* < 0.05). Regarding the *A. baumannii* strains, N-desmethyltamoxifen alone reduced the bacterial lung concentration and the mortality rate (−2.55 log_10_ CFU/g and −50%, *p* < 0.05) against the tigecycline-susceptible Ab#9 strain but showed no efficacy against the tigecycline-resistant Ab#186 strain. The combination of N-desmethyltamoxifen plus CMS reduced the bacterial lung and blood concentrations and the bacteraemia and mortality rates (−2.27 log_10_ CFU/g; −2.73 log_10_ CFU/mL, −70% and −40%; *p* < 0.05) of the tigecycline-resistant Ab#186 strain compared to CMS monotherapy. Regarding the combination of N-desmethyltamoxifen plus tigecycline, for the tigecycline-susceptible Ab#9 strain, the combination reduced the mortality rate (−45%, *p* < 0.05) and for the tigecycline-resistant Ab#186 strain, the combination reduced the lung and blood bacterial concentrations and the mortality rate (−3.27 log_10_ CFU/g; −4.95 log_10_ CFU/mL and −50%; *p* < 0.05) (Figure 5 and Supplementary Table S8).

The administration of N-desmethyltamoxifen alone in the pneumonia model, compared to the control group (Figure 5), reduced the mortality rate (−40%, *p* < 0.05) of the colistin-susceptible *E. coli* C1-7-LE strain, as well as the lung and blood bacterial concentrations (−2.86 log_10_ CFU/g and −5.88 log_10_ CFU/mL, *p* < 0.05) and the BSI and mortality rates (−40% and −50%, *p* < 0.05) of the colistin-resistant *E. coli* MCR-1+ strain. The combination of N-desmethyltamoxifen with CMS was better than CMS monotherapy only in relation to the reduction in the bacterial lung concentrations for the colistin-susceptible *E. coli* C1-7-LE strain (−1.19 log_10_ CFU/g, *p* < 0.05) and was better than tigecycline monotherapy in decreasing the mortality rate of the colistin-resistant *E. coli* MCR-1+ strain (−40%, *p* < 0.05). Regarding the *A. baumannii* strains, N-desmethyltamoxifen alone reduced the bacterial lung concentration and the mortality rate (−2.55 log_10_ CFU/g and −50%, *p* < 0.05) against the tigecycline-susceptible Ab#9 strain, without efficacy against the tigecycline-resistant Ab#186 strain. The combination of N-desmethyltamoxifen plus CMS reduced the bacterial lung and blood concentrations and the bacteraemia and mortality rates (−2.27 log_10_ CFU/g, −2.73 log_10_ CFU/mL, −70% and −40%, *p* < 0.05) of the tigecycline-resistant Ab#186 strain compared to CMS monotherapy. Regarding the combination of N-desmethyltamoxifen plus tigecycline, for the tigecycline-susceptible Ab#9 strain, the combination reduced the mortality rate (−45%, *p* < 0.05), and for the tigecycline-resistant Ab#186 strain, the combination reduced the lung and blood bacterial concentrations and the mortality rate (−3.27 log_10_ CFU/g, −4.95 log_10_ CFU/mL and −50%, *p* < 0.05) (Figure 5 and Supplementary Table S8).

## 3. Discussion

The results of the present study show that N-desmethyltamoxifen, the major tamoxifen metabolite, exhibited efficacy against a severe experimental pneumonia mouse model induced by *E. coli* and *A. baumannii* strains, especially in the case of the tigecycline-resistant Ab#186 strain, either alone or when combined with CMS or tigecycline, but also in isolation against the colistin-resistant *E. coli* MCR-1+ strain. The efficacy of the tamoxifen metabolite mix alone was restricted to only the colistin-susceptible *E. coli* C1-7-LE strain in the sepsis model, without demonstrable efficacy of any of the combinations when compared to the CMS or tigecycline monotherapies. However, the metabolite mix in combination with CMS or tigecycline decreased the bacterial concentrations in the lungs and blood against both tigecycline-susceptible and tigecycline-resistant *A. baumannii* strains. These results are in accordance with the bactericidal assays at the MICs, which were better than the in vitro bactericidal activity at C*_max_* in serum mice. 

In previous studies, tamoxifen garnered attention as a potential repurposed drug for the treatment of viral [30,31] and bacterial infections [32,33,34]. Additionally, clinical trials have explored tamoxifen’s utility in tackling other disorders [35,36]. Our previous work indicated that tamoxifen effectively combats *A. baumannii*, *Pseudomonas aeruginosa*, and *E. coli* infections through the regulation of the migration of immune cells from the bone marrow to the blood following bacterial infection [20]. Notably, these in vivo studies utilised a non-humanised dosage of 80 mg/kg [20,24] instead of the 40 mg/kg used in the present study.

Recent studies in the literature have shown that tamoxifen administered to mice induces transgenic murine models, with varying dosages up to 250 mg/kg [37,38]. However, analyses of PK/PD have revealed that a dose of 80 mg/kg in mice yields a plasmatic peak concentration significantly higher than that achieved in humans, following chronic tamoxifen treatment at 10 or 20 mg (0.120–0.122 mg/L) [23]. In the present study, we aimed to optimise the dosage to achieve a maximum plasmatic concentration within the 0.120–0.122 mg/L range. After a 40 mg/kg/ip dose, a maximum concentration of 0.159 mg/L was obtained. This humanised dosage did not induce toxicity effects, even though the cumulative three-day dose remained below the single-dose levels used in the prior literature [20,37].

However, a critical knowledge gap remains concerning the bactericidal activity and in vivo efficacy of the major circulating active metabolite of tamoxifen, N-desmethyltamoxifen, or the combination of all three metabolites against multidrug-resistant infections. To address this knowledge gap, two experimental murine models were investigated. Firstly, a peritoneal sepsis model demonstrated no efficacy, contrary to previous observations in our research group using higher dosages [24]. However, an optimised dosage may influence these outcomes, and the severe pathophysiology of the peritoneal sepsis murine model may obscure any differences between groups and treatments [39]. Consequently, we explored a model of localised infection—experimental pneumonia—which shed light on the efficacy of the metabolite mix and N-desmethyltamoxifen. In this model, CMS in combination with the metabolite mix reduced the bacterial concentrations in the lungs and blood for both *A. baumannii* and tigecycline-resistant strains. 

Tamoxifen primarily undergoes catabolism into N-desmethyltamoxifen, the major circulating metabolite [22]. For N-desmethyltamoxifen combined therapy for the experimental pneumonia murine model, we observed effectiveness against susceptible *E. coli* strains when combined with colistin and tigecycline. However, this combination did not yield any significant improvements for the colistin-resistant *E. coli* MCR-1+ strain. In tigecycline-resistant *A. baumannii*, CMS and tigecycline combined with N-desmethyltamoxifen decreased lung and blood bacterial concentrations and mortality rates. Furthermore, the colistin combination reduced BSI rates in the tigecycline-resistant *A. baumannii* strain. This enhanced efficacy of N-desmethyltamoxifen may be attributed to the adjusted dosage of 40 mg/kg, which achieved higher AUC_0–24_/MIC pharmacodynamic values for *A. baumannii* strains than for *E. coli* strains.

These PK values are also higher for N-desmethyltamoxifen compared with those reached with the combination of metabolites or tamoxifen. Also, the molecular action mechanism of tamoxifen could be involved in this bacterial clearance due to the modulation of the host immune system response [40]. In human beings, N-desmethyltamoxifen is metabolised to an active metabolite by the *CYP2D6* gene encoding the cytochrome P450 enzyme [22]. However, in the prokaryotic cells, the presence and number of copies of the *CYP2D6* gene are highly heterogeneous [41]. The *E. coli* genome does not encode the P450s cytochrome [42], while *A. baumannii* does in order to initiate biodegradation and metabolise xenobiotics, as described in the Kyoto Encyclopaedia of Genes and Genomes (KEGG) pathway database [43]. Higher levels of active metabolites could be the driver of greater efficacy in bacterial clearance.

Our study has certain limitations, which warrant further investigation. This study primarily focused on in vivo murine models, which may not fully represent human responses to tamoxifen metabolites. The lack of clinical data in humans limits the direct applicability of the findings to medical practice. Additionally, differences in the immune response between mice and humans could affect the outcomes and relevance of this study’s findings to human health. However, this study’s strengths lie in the utilisation of two experimental murine models to assess the efficacy of the metabolite mix and, specifically, N-desmethyltamoxifen in infections caused by *E. coli* or *A. baumannii*. Moreover, we have used well-characterised strains to confirm that they showed no differences in their pathogenicity and virulence. 

These findings have significant implications for clinical practice. We propose alternative combination therapies, particularly for difficult-to-treat infections caused by multidrug-resistant *A. baumannii*. Regarding the future, the next critical step involves conducting controlled and randomised clinical trials to assess whether N-desmethyltamoxifen-combined therapy may have a meaningful clinical impact on *A. baumannii* infections, thereby substantiating these promising preliminary results.

## 4. Materials and Methods

### 4.1. Tamoxifen, Tamoxifen Metabolites, and Antimicrobial Agents

For the in vitro assays, tamoxifen, N-desmethyltamoxifen, 4-hydroxytamoxifen, and antimicrobials were used as standard laboratory powders and were obtained from Sigma–Aldrich (Madrid, Spain), and endoxifen was purchased from MedChemExpress (Sollentuna, Sweden). Tamoxifen is water-insoluble, and to restore the standard laboratory powders, a corn oil/ethanol mixture was used. The mixture was vortexed and placed in a vacuum centrifuge for 30 min at 37 °C to evaporate the ethanol. The three tamoxifen metabolites are soluble in water. For the in vitro and in vivo studies, a mix of the three tamoxifen metabolites (metabolite mix, 33.3% each) was used, as well as N-desmethyltamoxifen alone. For the efficacy studies, clinical formulations were used: colistimethate sodium (CMS; Promixin, Bresso, Italy) and tigecycline (TIG; Normon, Madrid, Spain).

### 4.2. Bacterial Strains

Four well-characterised clinical strains, clonally unrelated [44,45,46], were used: *E. coli* C1-7-LE (ST8671, CC131), colistin- and tigecycline-susceptible; *E. coli* MCR-1+ (ST6108, CC405), colistin-resistant, tigecycline-susceptible, and MDR; *A. baumannii* Ab#9 (ST672, CC672), colistin- and tigecycline-susceptible; and Ab#186 (ST208, CC92), colistin-susceptible, tigecycline-resistant, and MDR (Table 1 and Supplementary Table S1) [47]. Because of the in vivo minimal lethal doses (MLD) of both *E. coli* and *A. baumannii* strains, pairs were different for the peritoneal sepsis (7.16 and 8.38 log_10_ CFU/mL for *E. coli* C1-7-LE and MCR-1+ strains, respectively, and 9.05 and 8.34 log_10_ CFU/mL for Ab#9 and Ab#186 strains, respectively) and pneumonia model characterisation (9.23 and 10.25 log_10_ CFU/mL for *E. coli* C1-7-LE and MCR-1+ strains, respectively, and 10.85 and 9.77 log_10_ CFU/mL for *A. baumannii* Ab#9 and Ab#186 strains, respectively); the in vitro competition indices [48], the adherence and invasion of eukaryotic cells [48], biofilm formation [48,49], and surface motility [50] were assessed to identify whether there were any different virulence traits between them (Supplementary Materials).

### 4.3. In Vitro Studies 

#### 4.3.1. Antimicrobial Susceptibility Testing

MICs were determined using a broth microdilution assay and interpreted according to the EUCAST 2024 breakpoints [51]. MBCs were also determined by sub-culturing onto blood agar plates (10 μL wells) containing antimicrobial concentrations greater than or equal to the MIC of the corresponding agent [52]. In vitro experiments were performed in triplicate and on different days. Moreover, MICs and MBCs were determined every six months to check the susceptibility stability.

#### 4.3.2. Time–Kill Assays 

The bactericidal and synergistic activities of the tamoxifen metabolites were evaluated using time–kill curves at MIC and C*_max_* (peak drug serum concentration in C57BL/6J healthy mice) after a single intraperitoneal (ip) dose of 40 mg/kg for the metabolite mix and N-desmethyltamoxifen. In brief, 20 mL of Mueller–Hinton broth was inoculated at approximately 5 × 10^5^ CFU/mL. Cultures were incubated at 37 ºC, and bacterial counts were determined by plating 100 μL of serial log 10 dilutions onto sheep blood agar plates at 0, 2, 4, 8, and 24 h. Bactericidal activity was defined as a ≥3 log_10_ decrease in the initial inoculum in CFU/mL and synergism as a ≥2 log_10_ decrease in bacterial concentration in comparison with the most active drug alone [28]. 

### 4.4. In Vivo Studies

#### 4.4.1. Animals

Immunocompetent C57BL/6J (JAX^®^ Strain) female mice, seven weeks old (Charles River Laboratories, Saint-Germain-Nuelles, France), were used. The animals had a murine pathogen-free sanitary status and were assessed for genetic authenticity. Mice were housed in an individually ventilated cage system, with ad libitum access to water and food. The study was carried out following the recommendations of the Guide for the Care and Use of Laboratory Animals [53]. The experiments followed the 2010/63/EU directive on the protection of animals used for scientific research. Experiments were approved by the Committee on the Ethics of Animal Experiments of the *Virgen del Rocío* University Hospital (0704-N-18, Seville, Spain) and the Andalusian Ministry of “Agricultura, Pesca y Desarrollo Rural” (31/07/2018/122). 

#### 4.4.2. Toxicity Studies

Before the efficacy studies, the metabolite mix and N-desmethyltamoxifen doses and regime schedule to be used in the efficacy studies were evaluated in healthy C57BL/6J female mice. Mice were monitored for 7 days, and no weight loss or changes in motility or indicative symptoms of pain/toxicity were observed.

#### 4.4.3. Pharmacokinetics (PK) and Pharmacodynamics (PD)

Serum levels were determined in groups of 30 healthy C57BL/6J female mice after a single dose of tamoxifen (40 mg/kg/ip), the metabolite mix (40 mg/kg/ip), and N-desmethyltamoxifen (40 mg/kg/ip). As previously described [28], in sets of three anaesthetised (thiopental, ip) mice, blood samples from the periorbital plexus were obtained at 0, 5, 10, 15, 30, 60, 120, 240, 480, and 1440 min after drug administration. Blood samples were immediately centrifuged (4500 rpm, 15 min at 4 °C), and serum samples were stored at −80 °C until drug concentration analysis using high-performance liquid chromatography (HPLC)–tandem mass spectrometry (LC-MS/MS). The C*_max_* of drugs, elimination half-life (t_1/2_), and area under the concentration–time curve from 0 to 24 h (AUC_0−24_) of each tamoxifen metabolite were calculated using the PKSOLVER program [54], as well as the PD variables AUC_0−24_/MIC, C*_max_*/MIC, and ΔT/MIC.

#### 4.4.4. Peritoneal Sepsis Model

A model widely used by our group was utilised [28]. In brief, groups of 10 and 5 mice for the different *E. coli* and the *A. baumannii* strains, respectively, were inoculated ip with 0.5 mL of a previously characterised MLD for each strain (Supplementary Materials). MLDs were 7.16 and 8.38 log_10_ CFU/mL for *E. coli* C1-7-LE and MCR-1+ strains, respectively, and 9.05 and 8.34 log_10_ CFU/mL for Ab#9 and Ab#186 strains, respectively. Mice were randomly selected for the following groups: (i) controls (infected without treatment); (ii) tamoxifen (40 mg/kg/qd/ip); (iii) the metabolite mix (40 mg/kg/qd/ip); (iv) CMS (20 mg/kg/tid/ip); (v) tigecycline (5 mg/kg/bid/sc); (vi) CMS + metabolite mix; and (vii) tigecycline + metabolite mix. Treatments started two hours after inoculation and lasted 72 h. Immediately after animal death or euthanasia (thiopental), spleen and blood samples were aseptically obtained and processed for quantitative cultures. 

#### 4.4.5. Pneumonia Model

A model widely used by our group was utilised [28]. In brief, groups of approximately 10 anaesthetised (ketamine/diazepam, ip) mice were inoculated intratracheally with 50 μL of the previously characterised MLD for each strain (Supplementary Materials). MLDs were 9.23 and 10.25 log_10_ CFU/mL for *E. coli* C1-7-LE and MCR-1+ strains, respectively, and 10.85 and 9.77 log_10_ CFU/mL for *A. baumannii* Ab#9 and Ab#186 strains, respectively. Mice were randomly selected for the same therapeutic groups detailed in the previous model; moreover, the following groups were evaluated in this model: N-desmethyltamoxifen (40 mg/kg/qd/ip); (ii) CMS + N-desmethyltamoxifen; and (iii) tigecycline + N-desmethyltamoxifen. Doses were the same as in the previous model and lasted the same length of time. Lung and blood samples were aseptically extracted and processed after animal death (or sacrifice; thiopental/ip) at the end of the study for quantitative cultures. 

### 4.5. Statistical Analysis

Mortality and positive blood cultures are expressed as proportions. Quantitative bacterial cultures of lungs, spleen, or blood (log_10_ CFU/g and log_10_ CFU/mL) are expressed as means ± SDs. Mortality and bloodstream infection (BSI) rates between groups were compared using chisquare or Fisher exact tests, with Bonferroni correction when appropriate. Quantitative variables were compared with ANOVA and Tukey and Dunnett post hoc tests. A *p* < 0.05 was considered significant. SPSS v24.0 software (SPSS Inc., Chicago, IL, USA) was used.

## 5. Conclusions

The results of this study highlight the potential of N-desmethyltamoxifen in combination with CMS or tigecycline as a new therapeutic strategy for MDR Gram-negative bacilli, especially *A. baumannii* infections, and they hold the potential to pave the way for future clinical trials and drive advancements in the management of these infections.

## Figures and Tables

**Table 1:** Strain multilocus sequence types and MIC/MBC (µg/mL) of colistin, tigecycline, tamoxifen, and tamoxifen metabolites for *Escherichia. coli* C1-7-LE (colistin-susceptible), *E. coli* MCR-1+ (colistin-resistant), *Acinetobacter baumannii* Ab#9 (tigecycline-susceptible), and *A. baumannii* Ab#186 (tigecycline-resistant) strains.

	MLST		MIC/MBC (mg/L)						
	ST	CC	COL	TIG	TAM	N-DTAM	HTAM	ENDX	MET
C1-7-LE	8671	131	0.12/0.12	0.12/0.12	>256/>256	128/256	256/256	64/128	4/4
MCR-1+	6108	405	**4**/8	0.5/0.5	>256/>256	128/256	256/256	128/256	4/4
Ab#9	672	672	0.06/0.12	0.12/0.12	>256/>256	16/16	256/256	32/32	4/8
Ab#186	208	92	0.25/0.5	**4**/4	>256/>256	64/64	256/256	32/64	4/4

ST: sequence type; CC: clonal complex. Tamoxifen metabolites (MET); colistin (COL); tigecycline (TIG); tamoxifen (TAM); N-desmethyltamoxifen (N-DTAM); hydroxytamoxifen (HTAM); endoxifen (ENDX). According to the European Committee on Antimicrobial Susceptibility Testing (EUCAST) clinical breakpoints v13.0 for *E. coli* and *A. baumannii*, they are susceptible ≤ 2 and resistant > 2 for colistin and susceptible ≤ 0.5 and resistant > 0.5 for tigecycline; currently, for *A. baumannii*, there are no tigecycline susceptibility criteria. For tigecycline, susceptible ≤ 0.5 and resistant > 0.5 were used as breakpoint criteria based on the manuscript of Pachón-Ibáñez, et al. [27]. Resistant strains are highlighted in bold.

**Table 2:** Pharmacokinetics parameters following the administration of a single intraperitoneal dose of colistimethate sodium, tigecycline, tamoxifen, and tamoxifen metabolites in healthy C57BL/6J female mice and pharmacodynamics for *Escherichia coli* C1-7-LE (colistin-susceptible), *E. coli* MCR-1+ (colistin-resistant), *Acinetobacter baumannii* Ab#9 (tigecycline-susceptible), and *A. baumannii* Ab#186 (tigecycline-resistant) strains.

Dose (mg/kg) and Administration Route	AUC_0–24_ (mg/L·min)	C*_max_* (mg/L)	T_1/2_ (min)	AUC_0–24_/MIC	C*_max_*/MIC
CMS (20, ip)	13.65	2.87	3.82	227.5 (C1-7-LE) 3.4 (MCR-1+) 227.5 (Ab#9) 54.6 (Ab#186)	
TIG (5, sc)	9.24	1.92	2.33	77 (C1-7-LE) 37.1 (MCR-1+) 77 (Ab#9) 2.31 (Ab#186)	
TAM (40, ip)	152.78	0.16	746.34	0.30 (C1-7-LE) 0.30 (MCR-1+) 0.30 (Ab#9) 0.30 (Ab#186)	3.1 × 10^−4^ (C1-7-LE) 3.1 × 10^−4^ (MCR-1+) 3.1 × 10^−4^ (Ab#9) 3.1 × 10^−4^ (Ab#186)
N-DTAM (40, ip)	572.52	1.16	432.65	4.4 (C1-7-LE) 4.4 (MCR-1+) 35.8 (Ab#9) 8.9 (Ab#186)	0.01 (C1-7-LE) 0.01 (MCR-1+) 0.07 (Ab#9) 0.02 (Ab#186)
N-DTAM (13.3, ip)	250.91	0.63	314.73	1.96 (C1-7-LE) 1.96 (MCR-1+) 15.68 (Ab#9) 3.92 (Ab#186)	4.9 × 10^−3^ (C1-7-LE) 4.9 × 10^−3^ (MCR-1+) 0.04 (Ab#9) 9.8 × 10^−3^ (Ab#186)
HTAM (13.3, ip)	130.28	0.98	231.91	0.51 (C1-7-LE) 0.51 (MCR-1+) 0.51 (Ab#9) 0.51 (Ab#186)	3.8 × 10^−3^ (C1-7-LE) 3.8 × 10^−3^ (MCR-1+) 3.8 × 10^−3^ (Ab#9) 3.8 × 10^−3^ (Ab#186)
ENDX (13.3, ip)	169.65	0.73	321.96	2.65 (C1-7-LE) 1.33 (MCR-1+) 5.30 (Ab#9) 5.30 (Ab#186)	0.01 (C1-7-LE) 5.7 × 10^−3^ (MCR-1+) 0.02 (Ab#9) 0.02 (Ab#186)

## Data Availability

Dataset available on request from the authors. The raw data supporting the conclusions of this article will be made available by the authors upon request.

## Supplementary Materials

The following supporting information can be downloaded at: https://www.mdpi.com/article/10.3390/antibiotics13050386/s1. Supplementary methods and results. Figure S1. In vitro bacterial growth and competition studies; Figure S2. Adherence and invasion in human lung A549 cells of *Escherichia coli*, colistin-susceptible (C1-7-LE); *E. coli*, colistin-resistant (MCR-1+); *Acinetobacter baumannii*, tigecycline-susceptible (Ab#9); and *A. baumannii* tigecycline-resistant (Ab#186) strains; Figure S3. Biofilm formation and surface motility of *Escherichia coli*, colistin-susceptible (C1-7-LE); *E. coli*, colistin-resistant (MCR-1+); *Acinetobacter baumannii*, tigecycline-susceptible (Ab#9); and *A. baumannii*, tigecycline-resistant (Ab#186) strains; Table S1. MICs of different antibiotics for *Escherichia coli*, colistin-susceptible (C1-7-LE); *E. coli*, colistin-resistant (MCR-1+); *Acinetobacter baumannii*, tigecycline-susceptible (Ab#9); and *A. baumannii* tigecycline-resistant (Ab#186) strains; Table S2. Primers of housekeeping genes to multi-locus sequence typing (MLST) of *Escherichia coli* and *Acinetobacter baumannii* strains; Table S3. Time–kill assays of colistin and/or tigecycline alone and combined with tamoxifen metabolites at MIC concentrations against *Escherichia coli* C1-7-LE (colistin-susceptible), *E. coli* MCR-1+ (colistin-resistant), *Acinetobacter baumannii* Ab#9 (tigecycline-susceptible), and *A. baumannii* Ab#186 (tigecycline-resistant); Table S4. Time–kill assays of colistin and/or tigecycline alone and combined with tamoxifen metabolite mix at the maximum mouse plasma concentration (C*_max_*) concentrations against *Escherichia coli* C1-7-LE (colistin-susceptible), *E. coli* MCR-1+ (colistin-resistant), *Acinetobacter baumannii* Ab#9 (tigecycline-susceptible), and *A. baumannii* Ab#186 (tigecycline-resistant); Table S5. Time–kill assays of colistin and/or tigecycline alone and combined with N-desmethyltamoxifen at the maximum mouse plasma concentration (C*_max_*) concentrations against *Escherichia coli* C1-7-LE (colistin-susceptible), *E. coli* MCR-1+ (colistin-resistant), *Acinetobacter baumannii* Ab#9 (tigecycline-susceptible), and *A. baumannii* Ab#186 (tigecycline-resistant); Table S6. Efficacy of colistimethate sodium and tigecycline monotherapies alone and their combinations with tamoxifen metabolites in the peritoneal sepsis model with *Escherichia coli* C1-7-LE (colistin-susceptible), *E. coli* MCR-1+ (colistin-resistant), *Acinetobacter baumannii* Ab#9 (tigecycline-susceptible), and *A. baumannii* Ab#186 (tigecycline-resistant); Table S7. Efficacy of colistimethate sodium and tigecycline monotherapies and their combinations with tamoxifen metabolites in the pneumonia model with *Escherichia coli* C1-7-LE (colistin-susceptible), *E. coli* MCR-1+ (colistin-resistant), *Acinetobacter baumannii* Ab#9 (tigecycline-susceptible), and *A. baumannii* Ab#186 (tigecycline-resistant); Table S8. Efficacy of colistimethate sodium and tigecycline monotherapies and their combinations with N-desmethyltamoxifen in the pneumonia model with *Escherichia coli* C1-7-LE (colistin-susceptible), *E. coli* MCR-1+ (colistin-resistant), *Acinetobacter baumannii* Ab#9 (tigecycline-susceptible), and *A. baumannii* Ab#186 (tigecycline-resistant). References [44,45,46,48,49,50] are cited in the supplementary materials.
