# Supplementary material for: Efficacy of Tamoxifen Metabolites in Combination with Colistin and Tigecycline in Experimental Murine Models of Escherichia coli and Acinetobacter baumannii
Source: Antibiotics (Basel). 2024 Apr 24;13(5):386. doi: 10.3390/antibiotics13050386 (PMC11117204; doi:10.3390/antibiotics13050386)
Supplement: Supplementary file 1 [file antibiotics-13-00386-s001.zip › antibiotics-2962653-supplementary.pdf]

METHODS

Multi-locus sequence typing

Primers for seven housekeeping genes (*adk*, *fumC*, *gyrB*, *icd*, *mdh*, *purA* and *recA*) for *E. coli* [44] and seven housekeeping genes (*gltA*, *gyrB*, *gdhB*, *cpn60*, *gpi*, *rpoD* and *recA*) for *A. baumannii* [45] were purchased from ThermoFischer (sequences detailed in Supplementary Table S1). The PCR amplification conditions were those previously described for *E. coli*[1] and *A. baumannii*.[45] PCR amplicons were gel-purified and sequenced using GFXTM PCR DNA and Gel Band Purification Kit (Cytiva, Uppsala, Sweden). Sequence types were determined by Sanger sequencing using an ABI3500 Genetic Analyzer (Applied Biosystems, Foster City, CA, USA) and the data were compiled through the website hosted at Enterobase (enterobase.warwick.ac.uk), following Achtman scheme for *E. coli* genes, and at pubMLST, following Oxford scheme for *A. baumannii* genes [46].

In vitro growth curves and competition indices

Each strain (5×10^5 CFU/mL) was grown in 10 mL of MHBII (Sigma-Aldrich, Spain) at 37 ºC. At different time points (2, 4, 8, 24 h) 100 µL aliquots were taken, and two-fold dilutions were plated on blood agar plates to calculate the corresponding CFU/mL. Competitive growth between the *E. coli* strains (C1-7-LE and MCR-1+) and the *A. baumannii* strains (Ab#9 and Ab#186) was assessed in MHB by mixing 5×10^5 CFU/mL each in the experiments. At the same previous time-points, aliquots from the cultures were seeded on both blood agar (Beckton Dickinson, USA) and Mueller–Hinton plates containing 2 mg/L of colistin in order to differentiate the colonies for both *E. coli* strains and 0.5 mg/L of tigecycline to separate the colonies for both *A. baumannii* strains [48]. Competition indices (CI) were defined as the number of resistant CFU

recovered/number of susceptible CFU recovered divided by the number of resistant CFU inoculum/number of susceptible CFU inoculum. If no colonies were recovered from cultures, the limit of detection of the assay (1 log_{10} CFU) was used to calculate competition indices. Experiments were performed in triplicate in different days.

Adherence and invasion of cultured human lung epithelial cells

Human lung epithelial cells, line A549 (ATCC® CCL-185™), were seeded (10^5 cells/well) for 24 h in 24-well plates. Strains were grown in Mueller Hinton broth (MHB; Sigma-Aldrich, Madrid, Spain) at 37 ºC during 20-24 hours, washed with phosphate-buffered saline (PBS; Sigma-Aldrich, Madrid, Spain), and resuspended in Dulbecco modified Eagle medium (DMEM; Biowest, Spain) prior to use in eukaryotic cell cultures. Before infection, cells were rinsed twice with PBS and then incubated with a 1:1000 dilution of an overnight culture of all the strains. Bacterial adherence and invasion assays were performed as previously described [48]. Assays were performed thrice in different days.

Biofilm assays

Biofilm production was measured as previously described [48,49]. Strains were cultured in MHB overnight at 37 ºC and then diluted to 10^6 CFU/mL in MHB. A total of 200 µL of the cell suspension was added to each well of a round-bottom 96-well plate and grown overnight at 37 ºC. Each well was washed twice to remove non-adherent bacteria, and 200 µL of 0.4 % crystal violet dye (Sigma-Aldrich, Spain) was added to each well. After incubation (10 minutes) at room temperature, the wells we washed twice and 200 µL of 96% ethanol added to each. After 15 min incubation at room temperature, biofilm formation was quantified by measuring the OD at 580 nm (Asys UVM 340 Microplate Reader, EE.UU.). *A. baumannii* ATCC 19606 and *A. baumannii* CR17 were

used as positive and negative controls, respectively. The results were normalized to the positive control strains, which were taken as 100%. Assays were performed in triplicate.

Surface motility assays

Surface motility was measured as previously described [50]. Overnight cultures of each strain were adjusted in PBS to an $OD_{600\ nm}$ of 0.6 (Lonza, Belgium). Three µL of the bacterial suspension was placed in the midpoint of a LB plate containing 0.3% agarose. Plates were incubated at 37 °C with 80% of humidity and the diameter of surface extension was measured at 24 hours of incubation. All assays were performed in triplicate.

Determination of minimum lethal doses (MLD)

1. Peritoneal sepsis murine model

Briefly, groups of 6 mice per inoculum were inoculated intraperitoneally (ip) with 0.5 mL of bacterial dilutions of the *E. coli* strains (C1-7-LE and *E. coli* MCR-1+,) and the *A. baumannii* strains (Ab#9 and Ab#186) starting from an inoculum of 10^4 CFU/mL approximately and ending with the first inoculum that cause 100% mice mortality (achievement MLD). Groups of 6 mice per inoculum were used, and survival rates were monitored during 7 days.

2. Pneumonia murine model

We performed the determination of the MLD for the same strains, also in groups of 6 animals per analysed inoculum starting with approximately 10^4 CFU/mL and until achieving the MDL as described before, with the difference of animals being anesthetized previously to in this case intra-tracheal inoculation (50 µL).

References

References cited in the Supplementary material are listed in the main manuscript.

RESULTS

1. *In vitro* Studies

Growth kinetics and competition indices

The growth kinetics of the *E. coli* and *A. baumannii* strains, when studied alone or in competition, were similar. The colistin-resistant *E. coli* MCR-1+ showed similar fitness than the colistin-susceptible C1-7-LE strain (CI: 1.18±0.03, 1.03±0.16, 1.06±0.04, 1.44±0.29 at 2, 4, 8, and 24 h, respectively), and the tigecycline-resistant Ab#186 strain fitness was similar compared to the tigecycline susceptible Ab#9 strain (CI: 0.99±0.04, 1.26±0.19, 0.64±0.43, 1.13±0.09 at 2, 4, 8, and 24 h, respectively) (Supplementary Figure S1).

Adherence and invasion of human lung epithelial cells

Results of virulence assays are showed in Figure 2. Colistin-resistant *E. coli* MCR-1+ presented higher adherence than C1-7-LE ($P<0.05$) and both resistant strains, *E. coli* MCR-1+ and *A. baumannii* Ab#186 showed higher cellular invasion ($P<0.05$) than the susceptible strains (Supplementary Figure S2).

Biofilm formation and surface motility assays

The biofilm formation was similar in the colistin-resistant *E coli* MCR-1+ and the tigecycline-resistant *A. baumannii* Ab#186 strains, compared to the susceptible counterparts. Similarly, the surface motility was similar in the resistant *E. coli* and *a. baumannii* strains (12.4±0.02 mm and 9.7±0.15 mm, respectively) than in the susceptible ones (11.1±0.05 mm and 7.85±0.07 mm) (Supplementary Figure S3).

Supplementary Figure S1. *In vitro* bacterial growth and competition studies.

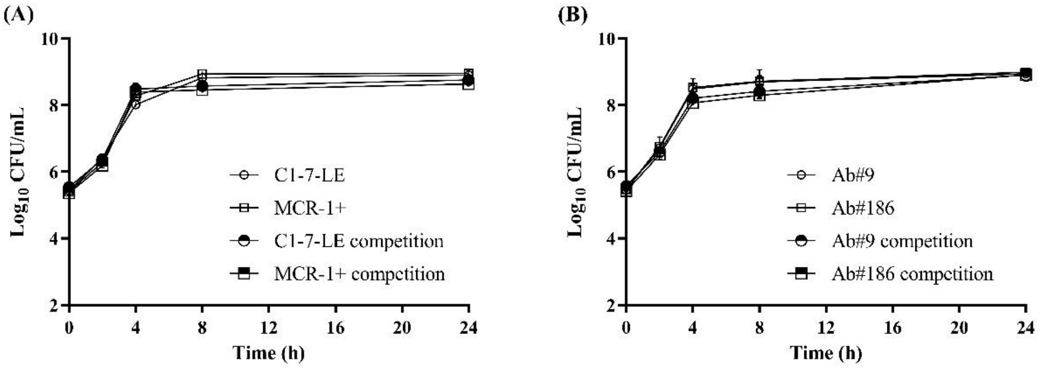

(A) *Escherichia coli* colistin-susceptible (C1-7-LE) and *E. coli* colistin-resistant (MCR-1+) and (B) *Acinetobacter baumannii* tigecycline-susceptible (Ab#9) and *A. baumannii* tigecycline-resistant (Ab#186) strains were grown in MHB alone and in competition. Data are represented as mean ± SD (n=3 replicates in different days).

Supplementary Figure S2. Adherence and invasion in human lung A549 cells of *Escherichia coli* colistin-susceptible (C1-7-LE), *E. coli* colistin-resistant (MCR-1+), *Acinetobacter baumannii* tigecycline-susceptible (Ab#9), and *A. baumannii* tigecycline-resistant (Ab#186) strains.

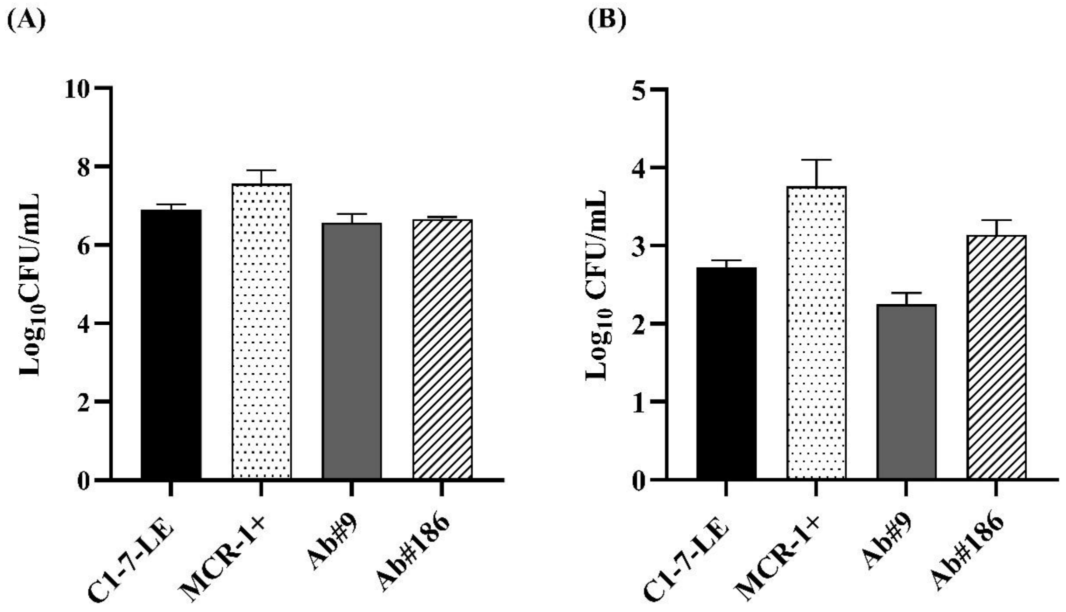

(A) Measurement of bacterial adherence two hours after infection with the *E. coli* and *A. baumannii* strains; (B) Measurement of bacterial invasion after 2 h infection. Data are represented as means ± SD (n = 3 replicates in different days).

**P*<0.05, U Mann-Whitney test.

Supplementary Figure S3. Biofilm formation and surface motility of *Escherichia coli* colistin-susceptible (C1-7-LE), *E. coli* colistin-resistant (MCR-1+), *Acinetobacter baumannii* tigecycline-susceptible (Ab#9), and *A. baumannii* tigecycline-resistant (Ab#186) strains.

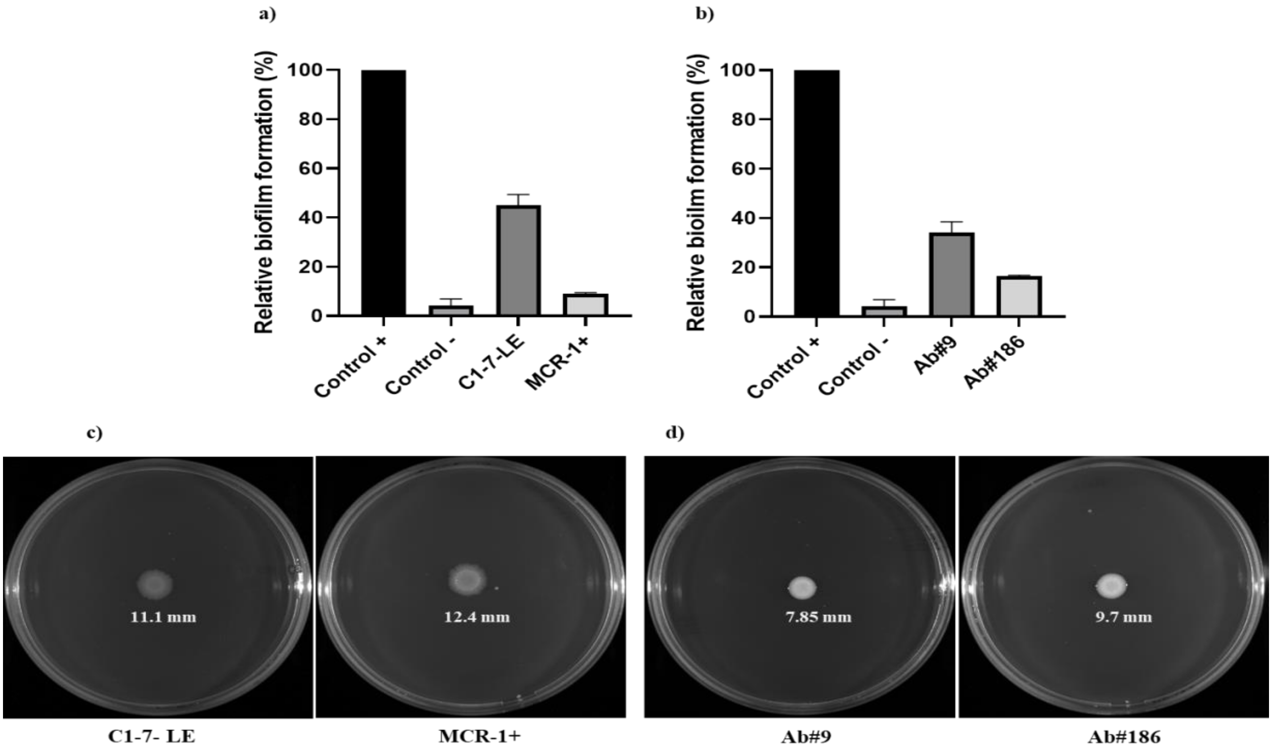

a) *E. coli* strains measurement of biofilm formation and b) *A. baumannii* strains measurement of biofilm formation; Positive control: *A. baumannii* ATCC 19606; Negative control: *A. baumannii* CR17; Data are represented as mean ± SD (n = 3 replicates in different days). c) *E. coli* surface motility and d) *A. baumannii* surface motility.

Supplementary Table S1. MIC of different antibiotics for of *Escherichia coli* colistin-susceptible (C1-7-LE), *E. coli* colistin-resistant (MCR-1+), *Acinetobacter baumannii* tigecycline-susceptible (Ab#9), and *A. baumannii* tigecycline-resistant (Ab#186) strains.

	**MIC** mg/L (S-I-R)			
	E. coli		*A. baumannii*	
**Antimicrobials**	C1-7-LE	MCR-1+	Ab#9	Ab#186
Ertapenem	<0.06 (S)	1 (R)	-	-
Meropenem	<0.06 (S)	<0.06 (S)	<0.5 (S)	16 (R)
Imipenem	0.25 (S)	>32 (R)	<0.5 (S)	32 (R)
Doripenem	-	-	≤0.001 (S)	8 (R)
Amikacin	8 (S)	8 (S)	<2 (S)	4 (S)
Gentamicin	1 (S)	2 (S)	<0.5 (S)	64 (R)
Tobramycin	-	-	<0.5 (S)	32 (R)
Netilmicin	-	-	<0.5 (*)	4 (*)
Piperacillin-tazobactam	4 (S)	1 (S)	-	-
Amoxicillin	0.5 (S)	4 (S)	-	-
Amoxicillin-clavulanic acid	0.5 (S)	8 (S)	-	-
Ceftazidime	0.12 (S)	16 (R)	16 (*)	>128 (*)
Cefotaxime	0.06 (S)	>32 (R)	-	-
Aztreonam	0.06 (S)	>32 (R)	-	-
Ciprofloxacin	<0.06 (S)	32 (R)	≤0.001 (S)	>64 (R)
Doxycycline	-	-	<0.5 (*)	32 (*)
Minocycline	-	-	<0.5 (*)	16 (*)
Tetracycline	-	-	1 (*)	>64 (*)
Tigecycline	0.12 (S)	0.5 (S)	0.12 (S)	4 (R)
Colistin	0.12 (S)	4 (R)	0.06 (S)	0.25 (S)
Rifampicin	-	-	<0.5 (*)	2 (*)

S: susceptible, standard dose regimen; ≤0.001: "susceptible, increased exposure"; R: resistant; *: there is insufficient evidence that the group is a good target for therapy with the agent,[7] As there is no tigecycline susceptibility criteria for *A. baumannii*, S ≤ 0.5 and R > 0.5 was used as breakpoint [8].

Supplementary Table S2. Primers of housekeeping genes to multi-locus sequence typing (MLST) of *Escherichia coli* and *Acinetobacter baumannii* strains.

E. coli strains		*A. baumannii* strains	
Gene	Sequence (5’- 3’)	Gene	Sequence (5’- 3’)
adk	F – ATTCTGCTTGGCGCTCCGGG<br>R – CCGTCAACTTTCGCGTATTT	*gltA*	F – AATTTACAGTGGCACATTAGGTCCC<br>R – GCAGAGATACCAGCAGAGATACACG
fumC	F – CACAGGTCGCCAGCGCTTC<br>R – GTACGCAGCGAAAAAGATTC	*gyrB*	F – TGTAAAACGACGGCCAGTGCNGGRTCYTTYTCYTGRCA<br>R – CAGGAAACAGCTATGACCAYGSNGGNGGNAARTTYRA
gyrB	F – TCGGCGACACGGATGACGGC<br>R – GTCCATGTAGGCGTTCAGGG	*gdhB*	F – GCTACTTTTATGCAACAGAGCC<br>R – GTTGGCGTATGTTGTGC
icd	F – TGGAAAGTAAAGTAGTTGTTCCGGCACA<br>R – GGACGCAGCAGGATCTGTT	*cpn60*	F – ACTGTACTTGCTCAAGC<br>R – TTCAGCGATGATAAGAAGTGG
mdh	F – TGAAAGTCGCAGTCCTCGGCGCTGCTGGCGG<br>R – TTAACGAACTCCTGCCCCAGAGCGATATCTTTCTT	*gpi*	F – AATACCGTGGTGCTACGGG<br>R – AACTTGATTTTCAGGAGC
purA	F – CGCGCTGATGAAAGAGATGA<br>R – CATACGGTAAGCCACGCAGA	*rpoD*	F – ACGACTGACCCGGTACGCATGTAYATGMGNGARATCGCNACNCT<br>R – ATAGAAATAACCAGACGTAAGTTNGCYTCNACCATYTGYTTYTT
recA	F – CGCATTCGCTTTACCCTGACC<br>R – TCGTCGAAATCTACGGACCGGA	*recA*	F – CCTGAATCTTCYGGTAAAAC<br>R – GTTTCTGGGCTGCCAAACATTAC

Supplementary Table S3. Time-kill assays of colistin and/or tigecycline alone and combined with tamoxifen metabolites at MIC concentrations against *Escherichia coli* C1-7-LE (colistin-susceptible), *E. coli* MCR-1+ (colistin-resistant), *Acinetobacter baumannii* Ab#9 (tigecycline-susceptible), and *A. baumannii* Ab#186 (tigecycline-resistant).

		E. coli		*A. baumannii*	
		**C1-7-LE**	**MCR-1+**	**Ab#9**	**Ab#186**
COL	2	-0.29	0.10	-1.04	-0.82
	4	-0.99	-1.16	-2.05	-1.41
	8	-1.86	-0.75	-1.11	-1.52
	24	3.02	3.18	3.04	3.00
TIG	2	0.07	1.24	0.09	-0.64
	4	0.63	1.25	0.32	-0.40
	8	1.04	3.74	1.12	-0.44
	24	3.40	4.27	3.07	-1.23
MET	2	-0.35	0.38	-0.67	-1.04
	4	-2.66	1.33	-1.07	-1.57
	8	-3.11	3.17	-2.12	-2.36
	24	2.88	4.14	3.13	3.00
COL + MET	2	-0.44	**-5.41**	-0.84	**-3.45**
	4	-3.37	**-5.41**	-3.08	**-4.56**
	8	-4.70	**-3.75**	**-4.74**	**-4.71**
	24	**-5.55**	**-2.33**	**-4.66**	**-3.50**
TIG + MET	2	-2.47	**-2.97**	-0.70	-2.92
	4	-3.45	**-3.52**	**-3.55**	**-4.33**
	8	-4.90	**-2.27**	**-4.59**	**-4.47**
	24	**-3.97**	**0.59**	**-3.74**	**-3.60**

Results are represented as differences (log_{10} CFU/mL) relative to the initial time-point (0 h). Green indicates a ≥3 log_{10} CFU/mL decrease. Bold, synergistic activity defined as ≥2 log_{10} decreases in bacterial concentration, respect to the most active drug alone at that time-point [9]. COL: colistin; TIG: tigecycline; MET: tamoxifen metabolites mix.

Supplementary Table S4. Time-kill assays of colistin and/or tigecycline alone and combined with tamoxifen metabolites mix at maximum mice plasma concentration (C_{max}) concentrations against *Escherichia coli* C1-7-LE (colistin-susceptible), *E. coli* MCR-1+ (colistin-resistant), *Acinetobacter baumannii* Ab#9 (tigecycline-susceptible), and *A. baumannii* Ab#186 (tigecycline-resistant).

		E. coli		*A. baumannii*	
		**C1-7-LE**	**MCR-1+**	**Ab#9**	**Ab#186**
COL	2	-2.66	-0.52	-1.78	-1.18
	4	-3.69	-0.15	-2.70	0.72
	8	-4.97	-0.12	-4.08	-1.64
	24	-3.25	3.72	-5.51	-0.93
TIG	2	0.24	0.33	-0.26	0.72
	4	0.04	1.77	-0.08	2.42
	8	-0.40	3.34	-2.93	3.19
	24	3.31	2.46	1.58	3.16
MET	2	0.81	0.78	0.75	0.55
	4	2.51	2.74	1.74	2.01
	8	3.62	3.74	3.30	3.32
	24	3.84	3.84	3.47	3.22
COL + MET	2	**-5.47**	-1.09	-1.92	-0.74
	4	-4.39	-1.54	-2.75	**-1.33**
	8	-4.97	-1.19	-2.86	-1.70
	24	0.27	3.76	-0.74	-1.17
TIG + MET	2	0.40	0.17	-0.28	0.71
	4	0.17	1.78	-0.46	1.90
	8	-0.46	2.84	0.11	3.14
	24	3.24	3.59	2.78	3.27

Results are represented as differences (log_{10} CFU/mL) relative to the initial time-point (0 h). Green indicates a ≥3 log_{10} CFU/mL decrease. Bold, synergistic activity defined as ≥2 log_{10} decreases in bacterial concentration, respect to the most active drug alone at that time-point [9]. COL: colistin; TIG: tigecycline; MET: tamoxifen metabolites mix.

Supplementary Table S5. Time-kill assays of colistin and/or tigecycline alone and combined with N-desmethyltamoxifen at maximum mice plasma concentration (C_{max}) concentrations against *Escherichia coli* C1-7-LE (colistin-susceptible), *E. coli* MCR-1+ (colistin-resistant), *Acinetobacter baumannii* Ab#9 (tigecycline-susceptible), and *A. baumannii* Ab#186 (tigecycline-resistant).

		E. coli		*A. baumannii*	
		**C1-7-LE**	**MCR-1+**	**Ab#9**	**Ab#186**
COL	2	-2.66	-0.52	-1.78	-1.18
	4	-3.69	-0.15	-2.70	0.72
	8	-4.97	-0.12	-4.08	-1.64
	24	-3.25	3.72	-5.51	-0.93
TIG	2	0.24	0.33	-0.26	0.72
	4	0.04	1.77	-0.08	2.42
	8	-0.40	3.34	-2.93	3.19
	24	3.31	2.46	1.58	3.16
N-DTAM	2	0.80	0.81	0.79	0.68
	4	2.59	2.64	1.89	2.12
	8	3.65	3.65	3.05	3.26
	24	3.72	3.95	3.55	3.16
COL + N-DTAM	2	-2.93	-0.30	-1.66	-0.89
	4	-4.17	-1.24	-2.75	**-1.36**
	8	-4.81	-1.31	-1.49	-1.98
	24	**-5.47**	3.91	-1.74	-0.42
TIG + N-DTAM	2	0.10	0.69	-0.14	0.67
	4	0.14	0.97	-0.62	2.06
	8	-0.10	2.46	-0.81	3.08
	24	3.28	3.64	2.33	3.23

Results are represented as differences (log_{10} CFU/mL) relative to the initial time-point (0 h). Green indicates a ≥3 log_{10} CFU/mL decrease Bold, synergistic activity defined as ≥2 log_{10} decreases in bacterial concentration, respect to the most active drug alone at that time-point [9]. COL: colistin; TIG: tigecycline; N-DTAM: N-desmethyltamoxifen.

Supplementary Table S6. Efficacy of colistimethate sodium and tigecycline monotherapies and their combinations with tamoxifen metabolites in the peritoneal sepsis model by *Escherichia coli* C1-7-LE (colistin-susceptible), *E. coli* MCR-1+ (colistin-resistant), *Acinetobacter baumannii* Ab#9 (tigecycline-susceptible), and *A. baumannii* Ab#186 (tigecycline-resistant).

Strains	Treatment groups (doses)	N	Log_{10} CFU/g spleen (mean ± SD)	Log_{10} CFU/mL blood (mean ± SD)	BSI (%)	Mortality (%)
	CTL	9	9.06 ± 0.70	8.86 ± 0.58[b]	100 (9/9)	89 (8/9)
	TAM (40 mg/kg/24h/ip)	5	9.80 ± 0.48	9.03 ± 0.68	100 (5/5)	100 (5/5)
	MET (40 mg/kg/24h/ip)	10	5.74 ± 2.07[a,b]	3.80 ± 3.44[a,b]	70 (7/10)	20 (2/10)[a,b]
C1-7-LE	CMS (20 mg/kg/8h /ip)	10	4.01 ± 1.73[a,b]	2.23 ± 2.05[a,b]	70 (7/10)	0 (0/10)[a,b]
	CMS+MET	10	4.03 ± 1.27[a,b,f]	1.07 ± 1.33[a,b]	50 (5/10)[a,b]	0 (0/10)[a,b]
	TIG (5 mg/kg/12h/sc)	11	2.04 ± 1.35[a,b,c]	0.22 ± 0.72[a,b]	14 (1/7)[a,b]	0 (0/11)[a,b]
	TIG+MET	11	2.16 ± 1.44[a,b,c,d]	0.57 ± 1.07[a,b]	43 (3/7)[a,b]	0 (0/11)[a,b]
	CTL	10	9.40 ± 0.42	8.67 ± 0.54	100 (10/10)	100 (10/10)
	TAM	5	9.55 ± 0.13	8.70 ± 0.77	100 (5/5)	100 (5/5)
	MET	10	9.06 ± 1.58	7.79 ± 2.02	100 (10/10)	90 (9/10)
MCR-1+	CMS	10	6.93 ± 4.41	6.16 ± 4.28	70 (7/10)	70 (7/10)
	CMS+MET	10	8.76 ± 1.78[f]	6.96 ± 2.31[f]	100 (10/10)	70 (7/10)
	TIG	11	2.73 ± 1.46[a,b,c]	0.00 ± 0.00[a,b,c,d]	0 (0/7)[a,b,c,d,e]	0 (0/11)[a,b,c,d,e]
	TIG+MET	11	3.59 ± 0.44[a,b,c,e]	0.54 ± 1.52[a,b,c,d,e]	13 (1/8)[a,b,c,d,e]	0 (0/11)[a,b,c,d,e]
	CTL	5	9.28 ± 0.07	8.40 ± 0.15	100 (5/5)	100 (5/5)
	TAM	5	9.64 ± 0.40	8.63 ± 0.29	80 (4/5)	80 (4/5)
	MET	5	9.96 ± 0.17[a]	8.60 ± 0.28	100 (5/5)	100 (5/5)
Ab#9	CMS	5	9.62 ± 0.30	8.17 ± 0.44	100 (5/5)	100 (5/5)
	CMS+MET	5	8.81 ± 0.76	7.19 ± 1.32[a,c,g]	100 (5/5)	100 (5/5)
	TIG	4	7.47 ± 0.18[a,b,c,d]	5.81 ± 0.23[a,b,c,d,e,g]	100 (4/4)	100 (4/4)
	TIG+MET	5	7.66 ± 0.21[a,b,c,d]	8.91 ± 0.36	100 (5/5)	100 (5/5)
	CTL	5	10.09 ± 0.54	9.60 ± 0.43	100 (5/5)	100 (5/5)
	TAM	5	10.01 ± 0.10	8.71 ± 0.16	80 (4/5)	80 (4/5)
	MET	5	10.21 ± 0.35	8.95 ± 0.37	100 (5/5)	100 (5/5)
Ab#186	CMS	5	3.31 ± 3.63[a,b,c,e]	3.32 ± 2.02[a,b,c]	100 (5/5)	20 (1/5)[a,c]
	CMS+MET	5	8.38 ± 2.56	6.71 ± 1.85	100 (5/5)	80 (4/5)
	TIG	5	5.18 ± 2.95[a,b,c]	4.96 ± 4.40	40 (2/5)	40 (2/5)
	TIG+MET	5	7.60 ± 2.42	7.35 ± 2.69	100 (5/5)	80 (4/5)

Quantitative bacterial cultures in spleen (log_{10} CFU/g) and blood (log_{10} CFU/ml) expressed as means ± SD. BSI: Bloodstream infection; ip: Intraperitoneal; sc: Subcutaneous; CTL: Control; TAM: Tamoxifen; MET: tamoxifen metabolites mix; CMS: colistimethate sodium; TIG: Tigecycline. [a] $P<0.05$ respect to CTL group; [b] $P<0.05$ respect to TAM group; [c] $P<0.05$ respect to MET group; [d] $P<0.05$ respect to CMS group; [e] $P<0.05$ respect to CMS+MET group; [f] $P<0.05$ respect to TIG group; [g] $P<0.05$ respect to TIG+MET group.

Supplementary Table S7. Efficacy of colistimethate sodium and tigecycline monotherapies and their combinations with tamoxifen metabolites in the pneumonia model by *Escherichia coli* C1-7-LE (colistin-susceptible), *E. coli* MCR-1+ (colistin-resistant), *Acinetobacter baumannii* Ab#9 (tigecycline-susceptible), and *A. baumannii* Ab#186 (tigecycline-resistant).

Strains	Treatment groups (doses)	N	Log_{10} CFU/g lungs (mean ± SD)	Log_{10} CFU/mL blood (mean ± SD)	BSI (%)	Mortality (%)
C1-7-LE	CTL	11	10.08 ± 0.15	8.73 ± 0.46	100 (11/11)	100 (11/11)
	TAM (40 mg/kg/24h/ip)	11	10.04 ± 0.19	8.19 ± 0.98	100 (11/11)	100 (11/11)
	MET (40 mg/kg/24h/ip)	11	9.41 ± 1.64	7.58 ± 2.54	91 (10/11)	91 (10/11)
	CMS (20 mg/kg/8h /ip)	10	6.45 ± 1.77[a,b,c]	1.93 ± 2.70[a,b,c]	50 (5/10)[a,b]	20 (2/10)[a,b,c]
	CMS+MET	11	3.78 ± 3.02[a,b,c]	1.06 ± 2.28[a,b,c]	27 (3/11)[a,b,c]	9 (1/11)[a,b,c]
	TIG (5 mg/kg/12h/sc)	10	4.88 ± 0.80[a,b,c]	0.41 ± 0.88[a,b,c]	20 (2/10)[a,b,c]	0 (0/10)[a,b,c]
	TIG+MET	11	3.64 ± 0.46[a,b,c,d,f]	0.35 ± 0.84[a,b,c]	18 (2/11)[a,b,c]	0 (0/11)[a,b,c]
MCR-1+	CTL	10	9.55 ± 0.57	8.23 ± 0.34	100 (10/10)	100 (10/10)
	TAM	11	8.87 ± 1.00	6.50 ± 1.88	100 (11/11)	91 (10/11)
	MET	11	8.46 ± 1.13	7.45 ± 1.65	100 (11/11)	73 (8/11)
	CMS	10	5.98 ± 0.76[a,b,c]	1.78 ± 2.32[a,b,c]	50 (5/10)[a,b,c]	0 (0/10)[a,b,c]
	CMS+MET	11	6.11 ± 2.64[a]	2.49 ± 3.71[a,c]	45 (5/11)[a,b,c]	18 (2/11)[a,b,c]
	TIG	10	4.34 ± 0.71[a,b,c,d]	0.36 ± 0.96[a,b,c]	40 (4/10)[a,b,c]	0 (0/10)[a,b,c]
	TIG+MET	11	4.04 ± 0.76[a,b,c,d]	0.80 ± 1.44[a,b,c]	27 (3/11)[a,b,c]	0 (0/11)[a,b,c]
Ab#9	CTL	11	10.17 ± 0.22	8.73 ± 0.47	100 (11/11)	100 (11/11)
	TAM	9	10.33 ± 0.21	9.03 ± 0.17	100 (9/9)	100 (9/9)
	MET	10	10.46 ± 0.26	8.95 ± 0.26	100 (10/10)	100 (10/10)
	CMS	11	6.49 ± 2.72[a,b,c]	3.57 ± 3.25[a,b,c]	73 (8/11)	45 (5/11)[a,b,c]
	CMS+MET	10	4.18 ± 1.09[a,b,c,f]	0.00 ± 0.00[a,b,c]	0 (0/10)[a,b,c,d,f]	0 (0/10)[a,b,c,d,f]
	TIG	11	7.61 ± 2.14[b,c]	3.52 ± 3.78[a,b,c]	55 (6/11)[a,b,c]	45 (5/11)[a,b,c]
	TIG+MET	10	5.76 ± 2.45[a,b,c]	2.37 ± 3.13[a,b,c]	40 (4/10)[a,b,c]	30 (3/10)[a,b,c]
Ab#186	CTL	11	10.05 ± 0.57	8.91 ± 0.43	100 (11/11)	100 (11/11)
	TAM	10	10.15 ± 0.32	8.65 ± 0.16	100 (10/10)	100 (10/10)
	MET	10	10.14 ± 0.34	8.60 ± 0.31	100 (10/10)	50 (5/10)[a,b]
	CMS	10	7.01 ± 0.58[a,b,c]	2.73 ± 2.27[a,b,c,f]	70 (7/10)	40 (4/10)[a,b]
	CMS+MET	9	4.71 ± 0.88[a,b,c,d,f]	1.17 ± 1.52[a,b,c,f]	44 (4/9)[a,b,c,f]	0 (0/9)[a,b,c,f]
	TIG	10	8.94 ± 1.46	7.31 ± 1.48	100 (10/10)	50 (5/10)[a,b]
	TIG+MET	10	5.66 ± 1.37[a,b,c,f]	2.80 ± 1.84[a,b,c,f]	80 (8/10)[a]	0 (0/10)[a,b,c,f]

Quantitative bacterial cultures in the lungs (log_{10} CFU/g) and blood (log_{10} CFU/mL), expressed as means ± SD. BSI: Bloodstream infection; ip: Intraperitoneal; sc: Subcutaneous; CTL: Control; TAM: Tamoxifen; MET: tamoxifen metabolites mix; CMS: colistimethate sodium; TIG: Tigecycline. [a] $P<0.05$ respect to CTL group; [b] $P<0.05$ respect to TAM group; [c] $P<0.05$ respect to MET group; [d] $P<0.05$ respect to CMS group; [e] $P<0.05$ respect to CMS+MET group; [f] $P<0.05$ respect to TIG group; [g] $P<0.05$ respect to TIG+MET group.

Supplementary Table S8. Efficacy of colistimethate sodium and tigecycline monotherapies and their combinations with N-desmethyltamoxifen in the pneumonia model by *Escherichia coli* C1-7-LE (colistin-susceptible), *E. coli* MCR-1+ (colistin-resistant), *Acinetobacter baumannii* Ab#9 (tigecycline-susceptible), and *A. baumannii* Ab#186 (tigecycline-resistant).

Strains	Treatment groups (doses)	N	Log_{10} CFU/g lungs (mean ± SD)	Log_{10} CFU/mL blood (mean ± SD)	BSI (%)	Mortality (%)
C1-7-LE	CTL	11	10.08 ± 0.15	8.73 ± 0.46	100 (11/11)	100 (11/11)
	TAM (40 mg/kg/24h/ip)	11	10.04 ± 0.19	8.19 ± 0.98	100 (11/11)	100 (11/11)
	N-DTAM (40 mg/kg/24h/ip)	10	8.14 ± 2.12	6.15 ± 2.24	100 (10/10)	60 (6/10)[a,b]
	CMS (20 mg/kg/8h /ip)	10	6.45 ± 1.77[a,b]	1.93 ± 2.70[a,b,c]	50 (5/10)[a,b,c,e,g]	20 (2/10)[a,b]
	CMS+N-DTAM	10	5.26 ± 0.44[a,b]	2.44 ± 0.38[a,b,c]	100 (10/10)	0 (0/10)[a,b,c]
	TIG (5 mg/kg/12h/sc)	10	4.88 ± 0.80[a,b,c]	0.41 ± 0.88[a,b,c,e,g]	20 (2/10)[a,b,c,e,g]	0 (0/10)[a,b,c]
	TIG+N-DTAM	10	5.18 ± 1.27[a,b]	2.95 ± 0.52[a,b,c]	100 (10/10)	0 (10/10)[a,b,c]
MCR-1+	CTL	10	9.55 ± 0.57	8.23 ± 0.34	100 (10/10)	100 (10/10)
	TAM	11	8.87 ± 1.00	6.50 ± 1.88	100 (11/11)	91 (10/11)
	N-DTAM	10	6.69 ± 1.53[a]	2.35 ± 2.19[a,b]	60 (6/10)[a,b]	50 (5/10)[a]
	CMS	10	5.98 ± 0.76[a,b]	1.78 ± 2.32[a,b]	50 (5/10)[a,b]	0 (0/10)[a,b,c]
	CMS+N-DTAM	10	5.55 ± 2.02[a,b]	2.41 ± 2.21[a,b]	60 (6/10)[a,b]	0 (0/10)[a,b,c]
	TIG	10	4.34 ± 0.71[a,b,c]	0.36 ± 0.96[a,b,c]	40 (4/10)[a,b]	0 (0/10)[a,b,c]
	TIG+N-DTAM	10	5.71 ± 2.31[a]	0.00 ± 0.00[a,b]	0 (0/10)[a,b,c,e,f]	0 (0/10)[a,b,c]
Ab#9	CTL	11	10.17 ± 0.22	8.73 ± 0.47	100 (11/11)	100 (11/11)
	TAM	9	10.33 ± 0.21	9.03 ± 0.17	100 (9/9)	100 (9/9)
	N-DTAM	10	7.62 ± 1.44[a,b]	6.57 ± 2.15	100 (10/10)	50 (5/10)[a,b]
	CMS	11	6.49 ± 2.72[a,b]	3.57 ± 3.25[a,b]	73 (8/11)	45 (5/11)[a,b]
	CMS+N-DTAM	10	5.96 ± 2.14[a,b]	2.23 ± 2.75[a,b,c]	50 (5/10)[a,b,c]	30 (3/10)[a,b]
	TIG	11	7.61 ± 2.14[b]	3.52 ± 3.78[a,b]	55 (6/11)[a,b,c]	45 (5/11)[a,b]
	TIG+N-DTAM	10	5.25 ± 1.74[a,b]	1.29 ± 1.37[a,b,c]	50 (5/10)[a,b,c]	0 (0/10)[a,b,c,d,f]
Ab#186	CTL	11	10.05 ± 0.57	8.91 ± 0.43	100 (11/11)	100 (11/11)
	TAM	10	10.15 ± 0.32	8.65 ± 0.16	100 (10/10)	100 (10/10)
	N-DTAM	10	9.25 ± 0.78	7.83 ± 1.61	100 (10/10)	80 (8/10)
	CMS	10	7.01 ± 0.58[a,b,c]	2.73 ± 2.27[a,b,c,f]	70 (7/10)	40 (4/10)[a,b]
	CMS+N-DTAM	10	4.74 ± 0.86[a,b,c,d,f]	0.00 ± 0.00[a,b,c,f,g]	0 (0/10)[a,b,c,d,f,g]	0 (0/10)[a,b,c,d,f]
	TIG	10	8.94 ± 1.46	7.31 ± 1.48	100 (10/10)	50 (5/10)[a,b]
	TIG+N-DTAM	10	4.67 ± 0.68[a,b,c,d,f]	2.36 ± 0.31[a,b,c,f]	100 (10/10)	0 (0/10)[a,b,c,f]

Quantitative bacterial cultures in the lungs (log_{10} CFU/g) and blood (log_{10} CFU/mL), expressed as means ± SD. BSI: Bloodstream infection; ip: Intraperitoneal; sc: Subcutaneous; CTL: Control; TAM: Tamoxifen; N-DTAM: N-desmethyltamoxifen; CMS: colistimethate sodium; TIG: Tigecycline. [a] P<0.05 respect to CTL group; [b] P<0.05 respect to TAM group; [c] P0.05 respect to N-DTAM group; [d] P<0.05 respect to CMS group; [e] P<0.05 respect to CMS+N-DTAM group; [f] P<0.05 respect to TIG group; [g] P<0. 05 respect to TIG+N-DTAM group.
